# Discrepancy in Screening Performances of Different Rapid Test Kits for SARS-CoV-2; a Letter to Editor

**DOI:** 10.22037/aaem.v9i1.1045

**Published:** 2020-12-13

**Authors:** Phee Kheng Cheah, Darlene F. Ongkili, Fatin Salwani Zaharuddin, Muammar Iqbal Hashim, Chiak Vun Ho, Heng Gee Lee, Phaik Kin Cheah

**Affiliations:** 1 Emergency and Trauma Department, Sabah Women and Children’s Hospital, Ministry of Health, Kota Kinabalu, Sabah, Malaysia.; 2 Emergency and Trauma Department, Queen Elizabeth Hospital, Ministry of Health, Kota Kinabalu, Sabah, Malaysia.; 3 Department of Pathology, Queen Elizabeth Hospital, Ministry of Health, Kota Kinabalu, Sabah, Malaysia.; 4 Infectious Disease Unit, Queen Elizabeth Hospital, Ministry of Health, Kota Kinabalu, Sabah, Malaysia.; 5 Faculty of Arts and Social Science, Universiti Tunku Abdul Rahman, Perak, Malaysia.

**Keywords:** Pandemics, public health, point-of-care systems, emergencies, coronavirus, diagnosis

**Dear Editor,**

Point-of-care testing has always been an attractive modality to quickly confirm diagnosis in the emergency department (ED). This attribute is highly valuable during the current Coronavirus Disease 2019 (COVID-19) pandemic caused by Severe Acute Respiratory Syndrome Coronavirus 2 (SARS-CoV-2), where early diagnosis means quicker case detection and earlier isolation. Rapid test kits (RTKs) developed to diagnose COVID-19 utilised two types of assay to detect SARS-CoV-2 infection(1). Molecular assays detect antigen in the form of viral RNA or protein on the patient’s respiratory tract, whilst serology immunoassays are used to detect IgM and IgG antibodies in the blood. There are many RTKs available commercially, but reports on effectiveness are scarce. We share the results of our study, which evaluated three colloidal gold-based immunoassay RTKs available in our centre (Sabah Women and Children’s Hospital, Kota Kinabalu, Malaysia).

We conducted an observational quantitative study in April 2020, after ethics approval was received from the Medical Research & Ethics Committee, Ministry of Health, Malaysia (Ethics approval number: NMRR-20-640-54491). The participants selected for this study were relatively well patients who were confirmed to be positive for COVID-19 using reverse transcriptase polymerase chain reaction (RT-PCR) testing on nasopharyngeal samples from contact screening. These patients were categorized based on their epidemiological link as well as if they were symptomatic or asymptomatic on their initial arrival to the ward. The three RTKs available in our centre were assigned as RTK1, RTK2 and RTK3. After obtaining consent, capillary sampling was performed and applied on the RTK. The result was read at 15 minutes as per kit’s instructions and recorded on the data collection form. The researchers then proceeded to complete the patient characteristics section and sampling details section of the data collection form. Patient characteristics included age, gender, comorbidities, date of admission, and patient presentation. Sampling details included date and result of RT-PCR test. Data were entered and analysed using Microsoft Excel Version 16.0. Since this study used detection of IgM and IgG antibodies, it is reasonable to determine the time gap between illness onset and result positivity. However, since onset of illness was unclear, time gap was defined as time between date of positive RT-PCR and the date RTK was conducted (PCR-RTK time gap).

In total, we tested 23 patients (52.17% female). RTK1 was tested on 11 patients. RTK2 and RTK3 were tested simultaneously on the same 12 patients (Table 1). Participants’ age for RTK1 ranged from 12 to 70 years, and for RTK2/ RTK3, it ranged from 14 to 58 years. About half of the total participants (12/23 or 52%) were asymptomatic on presentation. The PCR-RTK time gap for RTK1 ranged from 18 to 34 days (median 26 days), whilst for RTK2 and RTK3, it ranged from 13 to 38 days (median 25.5 days). 

Using RTK1, 5/11 (45.4%) patients produced positive IgG results, and 1/11 (0.1%) patients showed positive IgM result (Figure 1). More patients (10/12 or 83.3%) had positive IgG results using RTK3, but only one patient (0.08%) produced positive IgM result. Results were all negative for both IgM and IgG using RTK2. Only one patient tested positive for both IgM and IgG antibodies using RTK3. Both positive IgM results were at day 29 after RT-PCR result was positive. Fifty three percent (9/17) of positive RTK results were observed at day 21 to 30 after the diagnosis was confirmed by RT-PCR.

Based on our observations, patients tested IgG positive earlier using RTK3 compared to RTK1. Secondly, RTK2 did not produce positive results at all when RTK3 did. This reflects discrepancy in the performance of different RTKs. In China, one study tested patients using combined IgG and IgM RTK. The RTK was 88.7% sensitive and 90.6% specific (2). While these figures seemed promising, a study using RTK in an ED in Italy revealed a poor sensitivity of 18.4% and 91.7% specificity, making the RTK unreliable to diagnose COVID-19 (3). Possible explanation includes differences in antibody detection limit between RTKs, undetectable low antibody titre during early stages of infection, and individual immune response such as immunocompromised states (2-4). These individual factors may be the reason why some patients tested negative for both IgM and IgG antibodies up to 26 days after a positive RT-PCR result (Table 1).

The time gap for the majority of IgG positive patients was 11 to 30 days from positive RT-PCR results (Figure 1). This is an expected finding since IgG production is usually delayed. However, two patients tested IgM positive at 29 days. While it is generally understood that IgM is produced acutely and disappears two weeks after onset, research showed that in COVID-19 infection, IgM and IgG production peaks in the third and fourth week (5), which corresponded to the time frame of our patients’ results. This study is limited by the small sample size. Patient testing was based on convenience due to unpredictable kit availability, which only allowed researchers to use whichever kit was available in the laboratory at the time of testing. 

In conclusion, the RTKs tested in this study yielded inconsistent results. Health care providers and administrators should be aware that not all RTKs produce accurate and reliable results. This warrants standardisation and fine tuning of COVID-19 RTKs. We suggest that pilot testing be done prior to selecting an RTK for use to ensure that they produce intended results. In fact, at the time of writing, the RTK2 batch used was recalled by its manufacturer due to faulty product following complaints from end-user. As enticing as it may be, health care authorities must consider this diagnostic option carefully and wisely. 

**Table 1:** Results of three different rapid test kits (RTKs) of COVID-19 on 23 confirmed patients via Rt-PCR

**Age**	**Gender**	**Presentation**	Time Gap*	**RTK1**		**RTK2**		**RTK3**	
				IgM	IgG	IgM	IgG	IgM	IgG
52	Male	Symptomatic exposure	13			-	-	-	+
25	Female	Asymptomatic close contact	15			-	-	-	-
26	Female	Asymptomatic exposure	15			-	-	-	+
47	Female	Asymptomatic exposure	15			-	-	-	-
43	Female	Symptomatic exposure	15			-	-	-	+
48	Female	Asymptomatic exposure	16			-	-	-	+
53	Female	Symptomatic exposure	16			-	-	-	+
14	Male	Symptomatic close contact	18	-	-				
21	Female	Symptomatic close contact	19	-	-				
12	Male	Asymptomatic close contact	21	-	+				
33	Female	Asymptomatic close contact	21	-	+				
21	Male	Asymptomatic close contact	21	-	-				
58	Male	Symptomatic travel history	24	-	+				
21	Male	Asymptomatic mass gathering	24			-	-	-	+
28	Female	Symptomatic mass gathering	25	-	-				
70	Female	Asymptomatic close contact	25	-	+				
44	Female	Symptomatic travel history	26	-	-				
58	Male	Symptomatic travel history	27			-	-	-	+
61	Female	Asymptomatic close contact	29	+	-				
42	Male	Symptomatic exposure	29			-	-	+	+
14	Male	Asymptomatic mass gathering	32			-	-	-	+
65	Male	Asymptomatic close contact	34	-	+				
26	Male	Symptomatic mass gathering	38			-	-	-	+

*: Day.

**Figure 1:**
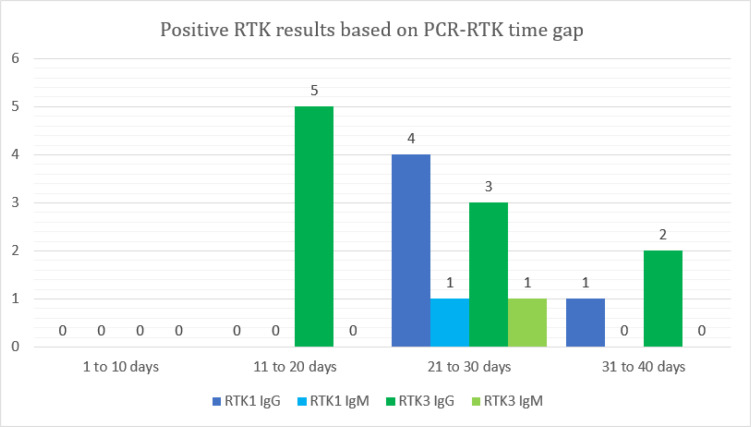
Positive results produced by RTKs based on the PCR-RTK time gap. Only positive RTK1 and RTK3 results were included in this chart. All results for RTK2 were negative and thus omitted from this chart

## Conflict of interest

None
